# Silencing P2X7 receptor downregulates the expression of TCP-1 involved in lymphoma lymphatic metastasis

**DOI:** 10.18632/oncotarget.5870

**Published:** 2015-11-06

**Authors:** Xudong Jiang, Wenjuan Mao, Ziyi Yang, Jia Zeng, Yi Zhang, Yang Song, Ying Kong, Shuangyi Ren, Yunfei Zuo

**Affiliations:** ^1^ Department of Clinical Biochemistry, College of Laboratory Diagnostic Medicine, Dalian Medical University, Dalian 116044, China; ^2^ Department of Surgery, the Second Affiliated Hospital of Dalian Medical University, Dalian 116023, China; ^3^ Department of Biochemistry, College of Basic Medical Sciences, Dalian Medical University, Dalian 116044, China

**Keywords:** P2X_7_R, TCP-1, lymphoma, proteomics, lymphatic-metastasis

## Abstract

P2X_7_R is an ATP-gated cation channel that participates in cell proliferation and apoptosis. TCP-1 assists with the protein folding. According to our previous research, the P2X_7_R has a potential role in P388D1 lymphoid neoplasm cells dissemination to peripheral lymph nodes. In order to make a further exploration about the probable mechanism, the lymph nodes which metastasized by P2X_7_R-silenced P388D1 cells or non-silenced cells were analyzed by 2DE and a MALDI-TOF-based proteomics approach. In the 64 proteins which were differentially expressed between two groups, TCP-1 was found to be significantly decreased in P2X_7_R shRNA group compared to controls. This correlation was also found in subsequent experiments *in vivo* and *in vitro*. The positive correlation between P2X_7_R and TCP-1 was also proved in both lymphoma and benign lymphadenopathy tissues from patients. It indicates that TCP-1 may be a crucial downstream molecular of P2X_7_R and plays a novel role in lymphoid neoplasm metastasis.

## INTRODUCTION

Lymphoma is one of the first discovered hematologic malignancies derived from lymph nodes or lymph tissues. Occurrence of lymphoma is often correlated with malignant proliferation of lymphocytes in immune responses. In China, Non-Hodgkin lymphoma (NHL) accounts for about 90% of malignant lymphadenoma (ML) and non-malignant lymphadenoma (NL) accounts for 10%. Worldwide, lymphomas developed in 566,000 people in 2012 and caused 305,000 deaths. They make up 3–4% of all cancers, making them as a group the seventh-most common form. In children, they are the third-most common cancer.

Biopsy of lymph node or bone marrow and histopathological analysis can not diagnose it accurately. Identification of novel molecule is crucial for lymphoma treatment and prognosis.

The P2X7 receptor (P2X_7_R) is expressed on the cell membrane; when activated by ATP, P2X_7_R forms a cation channel or a non-specific large pore, allowing Na^+^, K^+^, Ca^2+^ or other large hydrophilic molecules to pass through [1]. P2X_7_R is widely expressed on non-cancer cells, including hematopoietic stem cells, macrophages, mast cells, T-lymphocytes and B-lymphocytes [2], but it is often expressed on cancer cells, such as leukemia [3], prostate cancer [4], neuroblastoma [5] and thyroid papillary carcinoma [6]. Some studies have reported different levels of P2X_7_R associated with different levels of cancer severity and Zhang found P2X_7_R expression levels were different in different types of leukemia cells [3]. After one course of standard induction therapy, the survival rate in a high P2X_7_R expression group was lower than that in either a P2X_7_R negative group or a low P2X_7_R expression group. In B-cell chronic lymphocytic leukemia patients, Adinofi found the expression of P2X_7_R was significantly higher in lymphocytes from patients with the evolutive compared with the indolent variant [7]. These findings further suggest the expression of P2X_7_R is associated with the severity of cancer.

T complex polypeptide 1 (TCP-1) which acts as a member of the chaperonin assists the synthesis of cytoskeletal proteins, so far it has approved that TCP-1 is over-expressed in varieties of cancers and shows increased expression with advancing stage [8, 9]. As a member of the group II chaperonin family, can assist with the folding of newly synthesized proteins [10], including tubulins and actins [11], cyclin E [12], α-transducin [13] and von Hippel Lindau protein [14], which are all essential for cell functions. There are few reports of the relationship between TCP-1 and tumors; however, TCP-1 has been found to be correlated with the occurrence of liver cancer and colon cancer, and the content of TCP-1 was positively correlated with the severity of liver cancer and colon cancer [8]. Further, it has been reported that TCP-1 interacts with some tumor metastasis-associated proteins, such as tubulins and actins [11].

In our earlier study, in which we altered the expression of P2X_7_R in P388D1 cells and observed the changes in P388D1 metastatic capability, we found that the expression of P2X_7_R appeared to be correlated with P388D1 cells metastasis [15]. In this study, proteins from lymph nodes which metastasized by transfected P2X_7_R shRNA P388D1 cells and control cells were separated by two-dimensional gel electrophoresis (2DE). After that we analyzed the results of two groups by mass spectrum, TCP-1 appeared to have a close relationship with P2X_7_R in tumor metastasis. This results rouse our great interests and finally let us put TCP-1 into the focus of our study. As mass spectrometry (MS) results shown, TCP-1 expression was down-regulated in the metastatic lymph nodes of P2X_7_R shRNA DBA/2 mice than control group, indicating there might be correlation between the expression of TCP-1 and P2X_7_R. We silenced the expression of P2X_7_R in P388D1 cells with shRNA *in vivo* and *in vitro*. It was found that TCP-1 expression was also decreased significantly same as P2X_7_R. This result supported the indication that TCP-1 was correlated with P2X_7_R. Interestingly, we also found the positive relation between P2X_7_R and TCP-1 in human lymphoma tissues. In order to know if TCP-1 mediates the function that P2X_7_R inhibits the metastasis of murine lymphoid neoplasm cells to lymph nodes, we reduced the expression of TCP-1 in the P388D1, L5178Y and L1210 cell lines by TCP-1 shRNA and finally found that down-regulating TCP-1 could inhibit lymphoma cells metastasis *in vivo*.

In this study, we have proved that silence P2X_7_R expression can significantly decreased the expression of TCP-1 both *in vivo* and *in vitro*, the correlative expression of P2X_7_R and TCP-1 was also founded in human lymphoma tissues. In addition, we found that down-regulating TCP-1 can greatly attenuate the metastatic capability of the mouse lymphoma cells. Therefore, these results suggested that TCP-1 as a novel downstream molecular of P2X_7_R, played a crucial role in the inhibition of lymphoid neoplasm metastasis by silencing P2X7R. We propose that TCP-1 may be a new target for the therapy of lymphoma.

## RESULTS

### Proteins differentially expressed between the lymph nodes of P2X_7_R shRNA group and the controls

**Figure 1:**
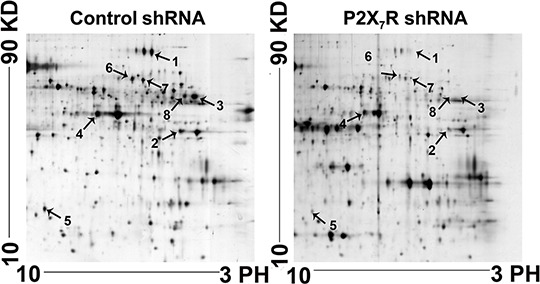
Proteomic analysis of the lymph nodes of P2X7R shRNA groups and control groups using 2-DE gels The arrows indicate protein spots that differ significantly between the P2X_7_R shRNA and control group. Relative spot intensity was calculated on the basis of the spot volume using Imagemaster™ 2D Platinum software. Only the spots indicated with arrows were used for identification.

On the basis of our earlier study, it was concluded that P2X_7_R has a potential role in tumor dissemination to peripheral lymph nodes, P388D1 cells (6 × 10^5^) transfected with P2X_7_R shRNA and P2X_7_R control shRNA were injected into the footpads of 6 mice respectively. The survival rate of mice treated by P2X_7_R shRNA was significantly longer than that of the control groups. The lymph nodes in the P2X_7_R shRNA group were much smaller than those in the control group [15]. In order to further study its mechanism, we got the lymph nodes by the same way as before, and the lymph nodes from P2X_7_R shRNA group and controls were analyzed by 2DE and staining with silver nitrate detected the protein spots in the gels within a pH range of 3 to 10. We selected 8 remarkable spots of differentially expressed protein between P2X_7_R shRNA group and the controls via statistical analysis of the 2DE gels (Figure 1).

### Mass spectrum identification of differentially expressed proteins

**Figure 2:**
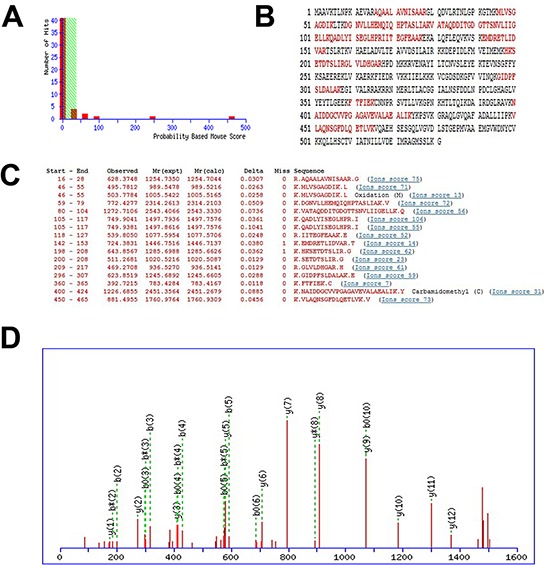
The identification of differentially expressed proteins **A, B, and C.** output of the database searching by the MASCOT engine using MS data used in the identification of TCP-1. The matched peptides were shown in bold red. **D.** MS/MS map marked with b ions and y ions for TCP-1 identification.

The 8 spots were identified by MS/MS analysis and 64 proteins were affirmed from these spots (Supplementary Table S1). Compared to these 64 proteins, we found 8 proteins with higher mass sequence coverage (> 30%): transferrin, an unnamed protein product (gi|74216774), fibrinogen β chain, hypothetical protein LOC433182, purine nucleoside phosphorylase, stress-induced phosphoprotein 1, phosphoglycerate kinase 1 and TCP-1. In contrast with TCP-1 the former 6 proteins were seldom correlated with tumor metastasis, and the phosphoglycerate kinase 1 participating mainly in the angiogenesis is critical for tumor expansion and metastasis [16]. Apart from the 7 proteins, TCP-1 displayed a significant decrease in P2X_7_R shRNA group. Yokota [8] found TCP-1 was correlated with liver cancer and colon cancer, and the level of TCP-1 was positively related with the severity of the cancer. TCP-1 can interact with a variety of proteins, including cytoskeletal proteins that impact on tumor metastasis [11]. Therefore, we focused on TCP-1 in our study. The mass spectra of TCP-1 in lymph nodes of P2X_7_R shRNA group and the P2X_7_R control shRNA group are shown in Figure 2. The MS/MS data supporting the suggestion appeared to be reliable (MASCOT score 461, sequence coverage 34%).

### Validation of the down-regulation of TCP-1 expression in P2X_7_R shRNA group *in vitro* and *in vivo*

**Figure 3:**
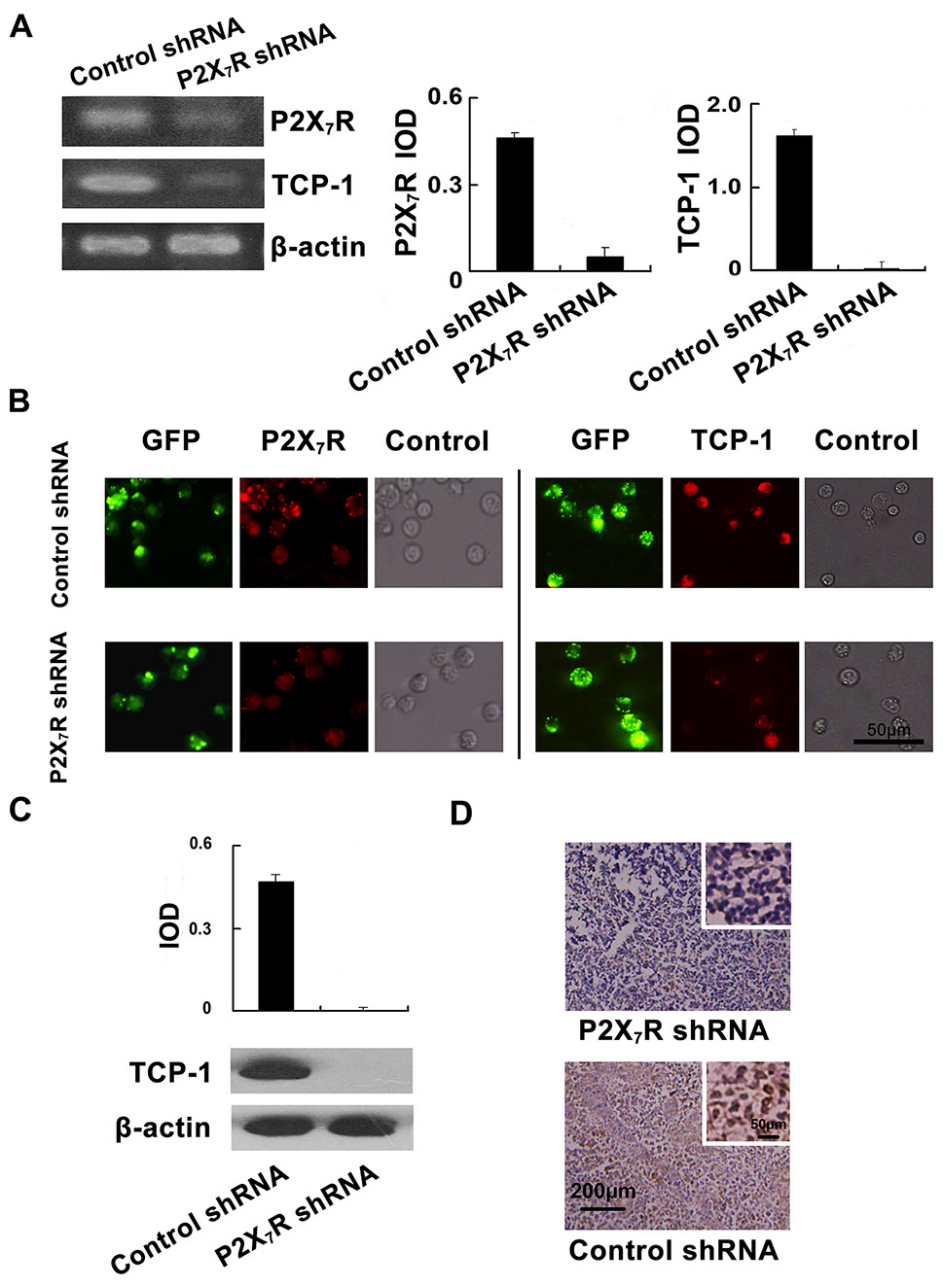
Validation of the down-regulation of TCP-1 expression in P2X7R shRNA group *in vitro* and *in vivo* **A.** P2X_7_R expression in P388D1 cells of P2X_7_R shRNA group and P2X_7_R control shRNA group was detected by RT-PCR. P2X_7_R as well as TCP-1 expressed in P388D1 cells in the P2X_7_R shRNA group was lower than that in the control group (Left). Densitometric analysis of the RT-PCR. Data were obtained using the Gel-Pro32 analyzer software with the values normalized to β-actin levels (Right). **B.** (Left Panel) P2X_7_R shRNA transfected cells and control cells were labeled with GFP; P2X_7_R was stained red with TRITC-labeled P2X_7_R antibody and located at the cell membrane. The expression of P2X_7_R in P388D1 cells of P2X_7_R shRNA group [10.4(±1.5) %, *n* = 4] was decreased compared to control group [72.6(±1.2) %, *n* = 4] (*p* < 0.001); The P388D1 cells in two groups were observed in bright field. (Right Panel) The transfection of P388D1 cells was visualized by GFP; P388D1 cells in two groups were incubated with TCP-1 goat polyclonal antibody and stained with TRITC-labeled secondary antibody. TCP-1 expressed in the cytoplasm of P388D1 cells was stained red. The expression of TCP-1 in P388D1 cells of the P2X_7_R shRNA group [9.8(±1.19) %, *n* = 4] were decreased compared to the controls [63.4(±1.2) %, *n* = 4] (*p* < 0.001); The P388D1 cells of the two groups were observed in bright field. Error bars represent ±S.D. of the mean. Scale bar represents 50 μm for all the micrographs. **C.** TCP-1 in the lymph nodes of the P2X_7_R shRNA group and control group was analyzed by Western blot. The corresponding quantification of the Western blot, values were normalized to β-actin levels. **D.** Immunohistochemical staining of TCP-1 in mouse lymph nodes of two groups. TCP-1 expressed in the cytoplasm of lymph nodes cells was stained brown.

The levels of P2X_7_R and TCP-1 in P388D1 cells transfected by P2X_7_R shRNA and P2X_7_R control shRNA were measured by RT-PCR and immunofluorescence respectively. As it shown, P2X_7_R shRNA simultaneously decreased the expression of P2X_7_R and TCP-1 in P388D1 cells compared to the controls (Figure 3A). Immunofluorescence analysis also demonstrated that lower of P2X_7_R and TCP-1 were expressed in P2X_7_R shRNA transfected P388D1 cells compared to the controls (Figure 3B). The differential expression of TCP-1 in DBA/2 mice lymph nodes of P2X_7_R shRNA group and control shRNA group was verified at the protein level by Western blot analysis with TCP-1 specific antibody (Figure 3C), we found TCP-1 expression in lymph nodes of P2X_7_R shRNA group was decreased significantly. Furthermore, immunohistochemistry also showed that TCP-1 expression was down-regulated in the lymph nodes of P2X_7_R shRNA group compared to the control shRNA group (Figure 3D). The results confirmed that lower levels of TCP-1 were expressed in the lymph nodes of P2X_7_R shRNA group than control group. These results further proved that silencing P2X_7_R can down-regulate the TCP-1 expression. It suggested, at least partly, the close relation between the expression of P2X_7_R and TCP-1.

### Analysis of the expression correlation between P2X_7_R and TCP-1 in lymphoma tissues from patients by immunohistochemistry

**Figure 4:**
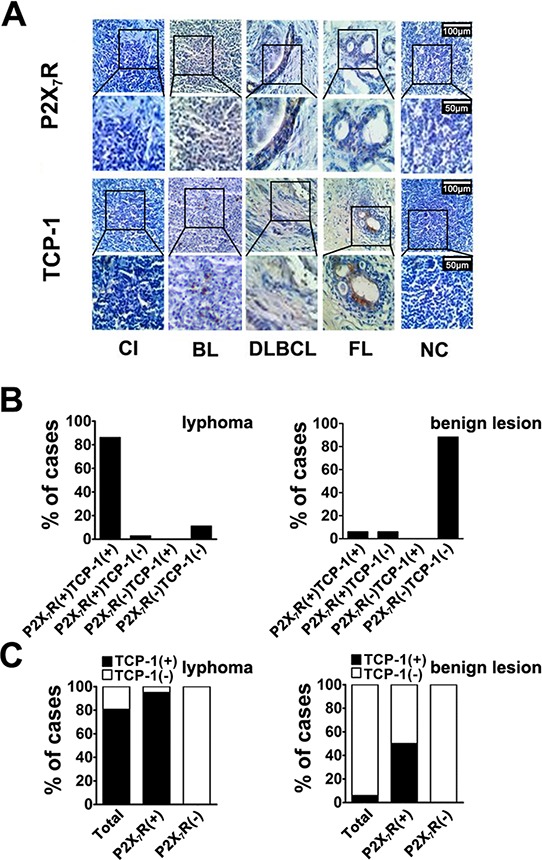
Immunohistochemical analysis of P2X7R and TCP-1 expression in human lymphoma and benign lymphadenopathy **A.** P2X_7_R and TCP-1 in various types of human lymphoma and benign lymphadenopathy was specifically detected by corresponding antibody. Sections came from a case of CI (Chronic inflammation), a case of BL (B cell lymphoma), a case of DLBCL (Diffuse large B cell lymphoma type), a case of FL (Follicular lymphoma) and a case of NC (Negative control). P2X_7_R and TCP-1 expression in same pathological specimen was observed by microscope, Scale bar represents 100 μm (upper plane) and 50 μm (bottom plane) respectively. P2X_7_R was stained brown on cell membrane and TCP-1 was shown in cytoplasm with brown. **B.** the different expression patterns of P2X_7_R and TCP-1 among all the lymphoma cases (Left); the different expression patterns of P2X_7_R and TCP-1 among benign lymphadenopathy (Right). **C.** the distribution of TCP-1 positive and negative cases among all lymphoma cases, P2X_7_R positive and negative lymphoma cases (Left); the distribution of TCP-1 positive and negative cases among all benign lymphadenopathy cases, P2X_7_R positive cases and negative benign lymphadenopathy (Right).

In order to know the relationship between P2X_7_R and TCP-1 in human lymphoma, immunohistochemical analysis about P2X_7_R and TCP-1 was performed. P2X_7_R and TCP-1 were observed expressing in both lymphoma and benign lymphadenopathy (Figure 4A). In lymphoma cases, the cases expressed P2X_7_R and TCP-1 (P2X_7_R+/TCP-1+) was more than 86%. In all the P2X_7_R positive cases, more than 96.8% expressed TCP-1. In benign lymphadenopathy, more than 88.2% cases had no expression of P2X_7_R and TCP-1 (P2X_7_R-/TCP-1-). In all the P2X_7_R positive cases, there is 50% expressed TCP-1 (Figure 4B and 4C). Details were shown in Table 1, According to Fisher exact test, the result suggested the positive correlation between P2X_7_R and TCP-1 in both lymphoma and benign lymphadenopathy of lymph nodes.

**Table 1:** The correlation of P2X_7_R and TCP-1 expression in lymphoma and benign lymphadenopathy

	Consistent results		Inconsistent results		*P* value^a^
	− / −	+ / +	− / +	+ / −	
Lymphoma P2X7R/TCP-1	8	62	0	2	*P* < 0.05
benign lymphadenopathy P2X7R/TCP-1	15	1	0	1	*P* < 0.05

a The *P* values were statistically analyzed in Fisher exact.

(+)stands for positive results: (−)stands for negative results.

### TCP-1 expressed in murine lymphoma neoplasm cells and could be silenced by TCP-1 shRNA

**Figure 5:**
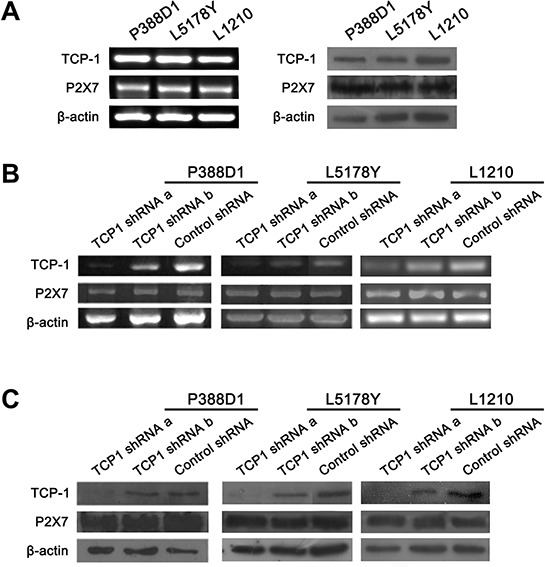
The expression of P2X7R and TCP-1 in different murine lymphoid neoplasm cell lines in TCP-1 shRNA group, control shRNA group and control group *in vitro* **A.** The expression of TCP-1 and P2X_7_R in P388D1, L5178Y and L1210 cell lines was detected by RT-PCR (Left) and western-blot assay (Right). **B.** the expression of TCP-1 and P2X_7_R in P388D1, L51778Y and L1210 cell line in different groups was detected by RT-PCR. **C.** TCP-1 and P2X_7_R expression in P388D1, L51778Y and L1210 cell line in different groups was detected by Western-blot assay.β-actin was used as control.

We detected the expression of TCP-1 in three kinds of murine lymphoma cell lines, P388D1, L5178Y and L1210, RT-PCR and Western blot confirmed that TCP-1 was indeed expressed in these cell lines (Figure 5A). All the cell lines were transfected by two different TCP-1 shRNA and control shRNA respectively. The effect of two TCP-1 specific shRNA was examined by RT-PCR and Western blot analysis (Figure 5B and 5C). Comparing with the control shRNA group, both of TCP-1 shRNA a and TCP-1 shRNA b could reduce the TCP-1 expression in the three cell lines, but TCP-1 shRNA a was more efficient than TCP-1 shRNA b. In TCP-1 shRNA a group. This data shown that the expression of TCP-1 was efficiently down-regulated in P388D1, L5178Y and L1210 cells transfected by TCP-1 shRNA a. Thus, TCP-1 shRNA a was chosen in the subsequent experiments.

### Experimental metastasis in DBA/2 mice

**Figure 6:**
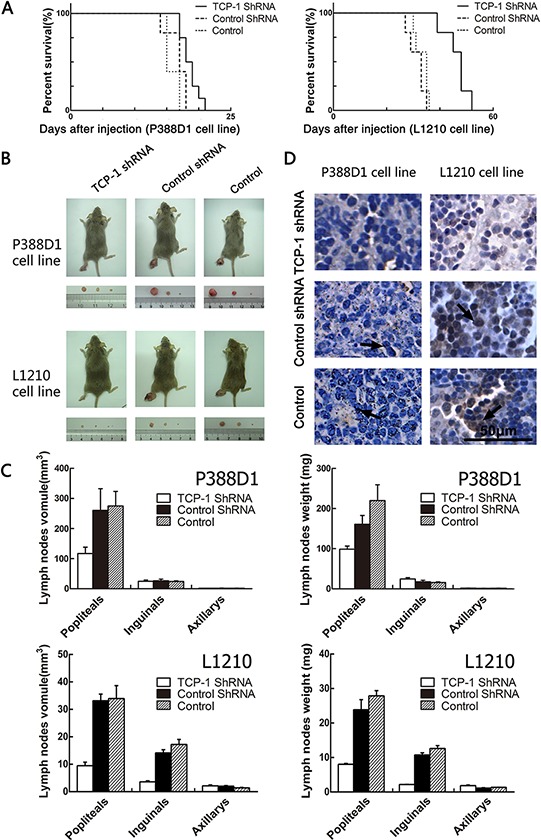
Experimental metastasis in DBA/2 mice with P388D1 and L1210 **A.** comparison of the survival percentage of the TCP-1 shRNA group, control shRNA group and control group (values were mean ± S.D., *n* = 5; *P* < 0.05). **B.** the lymph nodes got from the mice in TCP-1 shRNA group were significantly smaller than those in control shRNA group and control group. **C.** Reduced tumor volumes in the popliteal lymph nodes of DBA/2 mice injected with TCP-1 shRNA treatment P388D1 cells, compared with control shRNA treated (*P* = 0.0016) and non-treated P388D1 cells (*P* = 0.0016), and reduced tumor weight in the popliteal lymph nodes of DBA/2 mice injected with TCP-1 shRNA treatment P388D1 cells, compared with control shRNA treated (*P* = 0.0034) and non-treated P388D1 cells (*P* = 0.0026). Volumes of the popliteal lymph nodes of DBA/2 mice injected with TCP-1 shRNA treatment L1210 cells was significantly decreased compared to control shRNA treated (*P* = 0.000026) and non-treated L1210 cells (*P* = 0.0011), Volumes of the inguinal lymph nodes of TCP-1 shRNA treatment L1210 cells group was also significantly decreased compared to control shRNA treated group (*P* = 0.000029) and non-treated L1210 cells group (*P* = 0.000079); reduced tumor weight in the popliteal lymph nodes of DBA/2 mice injected with TCP-1 shRNA treatment L1210 cells, compared with control shRNA treated (*P* = 0.00069) and non-treated L1210 cells (*P* = 0.00013) and reduced tumor weight in the inguinal lymph nodes of DBA/2 mice injected with TCP-1 shRNA treatment L1210 cells, compared with control shRNA treated (*P* = 0.00016) and non-treated L1210 cells (*P* = 0.00022). Each datum point is the mean ± S.D. of five lymph nodes. **D.** immunohistochemical staining with TCP-1. Tissues came from lymph nodes metastasized P388D1 and L1210 cells respectively. TCP-1 was stained brown in lymph node tissues of three groups, Scale bar represents 50 μm for all the micrographs.

We have verified that TCP-1 shRNA a could silence the expression of TCP-1, and then we want to know whether the reduced expression of TCP-1 could regulate lymphoma progression and metastasis. For P388D1 cell line, 15 female and male inbred strain DBA/2 mice were divided at random into three groups (five mice in each group), TCP-1 shRNA a group, control shRNA group and control group. Each mouse was injected in the footpad with 6 × 10^5^ P388D1 cells. The same process was performed with L1210 cell line. Kaplan-Meier survival curves were constructed to compare the development of two cell lines of three groups. With P388D1 cell line, we found the survival percentage of mice in TCP-1 shRNA a group was conspicuous longer than it in control shRNA group and control group (*P* < 0.05), With L1210 cell line, the survival percentage of mice in TCP-1 shRNA a group was also longer than control shRNA group and control group (*P* < 0.01) (Figure 6A). Two cell lines formed tumor in all mice, in the comparison in three groups of each cell lines, we found the same results that lymph nodes of metastasized tumor cells from the mice in TCP-1 shRNA a group were smaller and lighter than those in the control shRNA and control group, no difference was found between control shRNA group and control group (Figure 6B and 6C). It was shown that down-regulated TCP-1 could reduce the metastasis of the tumor greatly. These results indicated that TCP-1 expressed in P388D1 and L1210 cells was necessary in the process that tumor metastasis to lymph nodes. To confirm this hypothesis, immunohistochemistry assay was performed to detected the expression of TCP-1 in the lymph nodes from three groups (Figure 6D). As expected, same results were got with two cell lines that TCP-1 was expressed lower in the TCP-1 shRNA group lymph nodes than other two groups. Based on these results, we concluded that TCP-1 played a crucial role in the inhibition of lymphoid neoplasm metastasis.

## DISCUSSION

Our results showed the metastatic capacity was decreased significantly in the P2X_7_R-silenced P388D1 cells. We found that the expression of TCP-1 in tumor metastatic lymph nodes was also decreased while P2X_7_R was silenced. These results supported our suggestion that TCP-1 is involved in lymphoma lymphatic metastasis. We have several suggestions to explain this phenomenon. First, TCP-1 assists the correct folding of the cytoskeletal proteins actin and tubulin [11], which are important components of the filaments and microtubules needed for cell movements. A decreased level of TCP-1 reduces actin and tubulin synthesis, thus reducing the amount of cell motility-related proteins and weakening tumor metastatic potential [17]. Second, studies have shown TCP-1 can influence the synthesis of cell-cycle proteins, such as cyclin E, which has been reported to be highly expressed in many types of cancer cells and to regulate the cell cycle [18, 19]. A decreased level of TCP-1 lowers the content of cyclin E, which reduces the capability of tumor cell division and proliferation and, thus, reduces tumor metastasis. Finally, TCP-1 can interact with other proteins, such as the von Hippel-Lindau (VHL) tumor suppressor protein [14], histone deacetylase [20] and protein phosphatase PP2A regulatory subunit B [21], myosin and transducin. In mammalian cells, the unassembled form of VHL is associated with two different molecular chaperones, Hsp70 and TCP-1 [14, 22]. *In vitro* translation experiments in TCP-1 immunodepleted extracts indicate that this chaperonin is required for VHL incorporation into von Hippel-Lindau protein (VHL)-elongin BC [22]. More than 70 unidentified polypeptide species associated with TCP-1 *in vivo* can be immunoprecipitated [10].

In addition, the P2X_7_R has been shown to mediate ATP-induced cell death in human embryonic kidney cells [23] and human cervical epithelial cells [24], and its downstream signaling is coupled to pro-inflammatory cascades [25]. The P2X_7_R-mediated apoptosis of mast cells involved the activation of caspases −3 and −8, and the cleavage of caspase-3 substrate PARP [26]. It has been suggested that in the time period between commitment to apoptosis and actual cell death, agonistic stimulation of P2X_7_R also activates additional signaling pathways, which may lead to cytokine production in case of proteolytic processing and release of IL-1 from LPS-primed macrophages that precedes cell death [27], and activation of various transcription factors like NFAT or NF-κB [28]. Brief stimulation of the P2X_7_R with ATP results in formation of a non-selective cationic channel that promotes the influx of Ca^2+^ and the equilibration of the transmembrane sodium and potassium gradients leading to membrane depolarization [29]. Extracellular release of active IL-1β and IL-18 is dependent on ATP-sensitive P2X_7_R activation [30]. The anti- apoptotic property of the P2X_7_R has been described in neutrophils of patients with rheumatoid arthritis (RA) [31]. Serum amyloid A (SAA) purified from the plasma of patients with RA, or recombinant SAA, suppressed both spontaneous and α-FAS (CD95) induced-neutrophil apoptosis of human neutrophils *in vitro*; Oxidized ATP is an unselective P2X_7_R antagonist and it can attenuate inflammatory responses independent of P2 receptor blockade and inhibit this SAA-mediated anti-apoptotic effect [30]. Nagaoka et al. have also demonstrated an anti-apoptotic action of P2X_7_R [32]. So this anti-apoptotic action was reduced in the P2X_7_R-silenced P388D1 cells, it resulted a promotion of apoptosis, thus reduced tumor metastasis. Although no direct association with cancer metastasis has been found, these proteins have close relationships with gene expression, cell signal transduction and the cell cycle; therefore, TCP-1 affecting the synthesis of these proteins is likely to influence tumor metastasis.

The related mechanism of P2X_7_R and TCP-1 may be very complex, possibly involving signaling pathway, cytokine and other multilevel regulation. There are several signal pathways which may be associated with it. Recent studies found activation of the MAPK pathway in cancer cells can enhance cell invasion and promote peripheral vascular generation, thereby increasing the metastatic potential of cancer [33]. It has been reported that inhibiting P2X_7_R expression can suppress activation of the MAPK pathway [34, 35]. Inhibition of the MAPK pathway can reduce the synthesis of ternary complex factors (TCFs), which further inhibit expression of the TCP-1 gene [36]. PI3K is also a possible pathway. Activation of the PI3K pathway can promote transformation of normal cells into abnormal calls, and can enhance cancer cell invasion and metastatic potential [37]. AKT is the important intermediate molecule in the PI3K pathway. It has been reported that inhibition of P2X_7_R expression could inhibit activation of the PI3K pathway and reduce the level of AKT phosphorylation [38]. TCP-1 is the downstream effector of AKT and decreased AKT phosphorylation reduces the level of TCP-1 phosphorylation [39]. But this demonstrated only that inhibition of P2X_7_R causes a decrease of TCP-1 phosphorylation; whether it changes TCP-1 content is still not known. Besides, caspase as an important apoptosis pathway is closely related with P2X_7_R. Studies have found in skin neoplasias of mice the activation of P2X_7_R can activate caspase-9 and caspase-3 so as to activate the mitochondrial - caspase-9 apoptosis pathway [40]; the study of TCP-1 also found that in uterine cancer cells the disruption of TCP-1 activates both intrinsic (caspase-3, caspase-6, caspase-7, and caspase-9) and extrinsic (caspase-2,caspase-8, and caspase-10) caspase-dependent apoptotic pathway [41]. These to some extent manifested the apoptosis pathway is likely to be linked to P2X_7_R and TCP-1.

## MATERIALS AND METHODS

### Ethics statement

Investigation has been conducted in accordance with the ethical standards and according to the Declaration of Helsinki and according to national and international guidelines and has been approved by the Research Ethics Committee of Dalian Medical University in agreement with institutional guidelines.

### Patients

Between 2003 and 2011, a total of 89 consecutive patients, include 72 lymphoma and 17 benign lymphadenopathy, at the 2^nd^ Affiliated Hospital of Dalian Medical University were involved in the study. The histological types were as follows: in 72 lymphoma, there were B-cell lymphoma (*n* = 17), T-cell lymphoma (*n* = 9), diffuse large B-cell lymphoma (*n* = 28), follicular lymphoma (*n* = 10), Hodgkin's lymphoma (*n* = 6), mantle cell lymphoma (*n* = 1), small lymphocytic lymphoma (*n* = 1). 17 benign lymphadenopathy include chronic inflammation (*n* = 8), reactive hyperplasia (*n* = 9). Informed consent was obtained from all participants. Clinic pathological characteristics of patients was shown in Table 2. More detail information of patients could be seen in Supplementary Table S2.

**Table 2:** Clinic pathological characteristics of patients

Characteristic	Patients	%
**Lymphoma**	72	
Age Median (range)	56.5 (20–18)	
Mean ± SD	56.29 ± 14.66	
Gender		
Male	42	58.3
Female	30	41.7
Histologic type		
B-cell lymphoma	17	23.6
T-cell lymphoma	9	12.5
Diffuse large B-cell lymphoma	28	38.9
Follicular lymphoma	10	13.9
Mantle cell lymphoma	1	1.4
Small lymphocytic lymphoma	1	1.4
Hodgkin's lymphoma	6	8.3
**Benign lymphadenopathy**	17	
Age Median (range)	47 (4–75)	
Mean ± SD	44.65 ± 21.3	
Gender		
Male	8	47.1
Female	9	52.9
Histologic type		
Chronic inflammation	8	47.1
Reactive hyperplasia	9	52.9

### Cell culture and mice

Three murine lymphoma cell lines were used in this study, P388D1, L5178Y and L1210 respectively. All the cell lines were purchased from the Institute of Biochemistry and Cell Biology, the Chinese Academy of Sciences (Shanghai, China). P388D1 cells were cultured in RPMI 1640 medium (Sigma) supplemented with 10% FBS and 100 U/ml penicillin/streptomycin (Beyotime) at 37°C in a humidified 5% CO2 atmosphere. L5178Y and L1210 cells were cultured in DMEM medium (Sigma) supplemented with 10% FCS and 100 U/ml penicillin/streptomycin (Beyotime) at 37°C in a humidified 5% CO2 atmosphere. 6–8 weeks old DBA/2 mice were obtained from the specific pathogen free animal center of Dalian Medical University (Dalian, China).

### Plasmids and lentivirus

shRNA sequence for knockdown of P2X_7_R was 5′-UGAGCGAUAAGCUGUACCAUUGAAGAAGUGG UACAGCUUAUCGCUCAUU-3′. The control shRNA sequence for P2X_7_R was 5′-GTTCTCCGAACGTGTCAC GTCAAGAGATTACGTGACACGTTCGGAGAATT-3′. Two different sequences for silencing TCP-1 were a: 5′-GCTGGAGACATCAAACTTACT-3′; and b: 5′-GCGATGGCACTACATCCAATG-3′. A scrambled sequence having no significant homology with target sequence databases as negative control. It was 5′-TTCTCCGAACGTGTCACGTTTC-3′. Five plasmids contained P2X_7_R shRNA, P2X_7_R control shRNA, two kinds of TCP-1 shRNA, and TCP-1 control shRNA respectively and three lentivirus contained two kinds of TCP-1 shRNA, and TCP-1 control shRNA were constructed by Shanghai Gene Pharma Co. Ltd.

### Stable transfection of cells

For plasmids transfection, P388D1 and L5178Y cells were plated at 40–50% confluence in 3.5 cm cell culture dishes and kept overnight. TCP-1 shRNA and control shRNA were transfected with Effectene Transfection Reagent (Qiagen, Cat. No. 301425) following the manufacturer's instructions. For lentivirus infection, the most suitable multiplicity of infection (MOI) and the minimum concentration of puromycin (Sigma, Cat. No. P8833) required to kill uninfected cells were determined by preliminary test. L1210 cells in logarithmic growth phase were seeded in 24 wells culture plates with 5 × 10^4^ cells/well. Since grown to 30% confluence, cells were infected with lentivirus at a MOI of 60 with polybrene (10 μg/mL). After 24 h, the medium containing virus were replaced with fresh complete medium. Stably transfected L1210 cells were selected by adding puromycin (4 μg/mL) in medium. GFP in the successfully transfected cells was verified by fluorescent microscope and the transfection efficiency was measured by the percentage of GFP positive cells. The silence efficiency was evaluated by RT-PCR and western blot analysis.

### Animal experiments

The lymph node tissues were obtained as our earlier described [15]. Briefly, transfected murine lymphoma cells (6 × 10^5^) were injected into the footpads of DBA/2 mice in different groups respectively. The mice were sacrificed after several weeks and the lymph nodes were collected and prepared for subsequent experiments.

### Two-dimensional gel electrophoresis (2DE) and image analysis

The lymph node tissue sample (0.1 g) from mice injected by P3388D1 treated by P2X7R shRNA and control shRNA was ground into a powder in liquid nitrogen, homogenized in 1 ml of lysis buffer (40 mM Tris-HCl, 7 M urea, 2 M thiourea, 4% Chaps, 1% DTT, 1 mM EDTA) on ice, vortex mixed thoroughly, kept on ice for 1 h then centrifuged at 15,000 g for 1 h at 4°C. The supernatant was collected and placed into an Eppendorf tube. After preparation of the total protein, isoelectric focusing (IEF) was done according to the guide provided with the IPGphor system (Amersham Pharmacia Biotech). The IEF gel strips were equilibrated for 15 min in 10 ml of equilibrium solution (50 mM Tris-HCl, pH 8.8, 30% glycerol, 6 M urea, 2% SDS and a trace of bromophenol blue). After equilibration, separation in the second dimension was done by SDS-PAGE (13% polyacrylamide gel) using the PROTEN II xi Cell system (BIO-RAD) and then the gel was stained with silver nitrate. The protein spots were detected, quantified and matched using ImageMaster 2D Platinum (Amersham Pharmacia Biotech).

### In-gel tryptic digestion and protein identification by MS

After 2DE, the sample was subjected to in-gel tryptic digestion and protein identification by MS. Briefly, protein spots of interest were excised and destained. In-gel digestion was done with 0.01 μg/μl trypsin for 15 h at 37°C. The tryptic peptides were extracted from the gel and dried by centrifugal lyophilization. The dried peptide mixture was dissolved in 5% trifluoroacetic acid (TFA). Electrospray ionization (ESI-MS/MS) was carried out with a hybrid quadrupole orthogonal acceleration tandem mass spectrometer (Q-TOF2, Micromass Ltd., Manchester, UK). Glu-Fibrinopeptide was used to calibrate the instrument in the MS/MS mode. MS/MS spectra were transformed using MaxEnt3 (MassLynx, Micromass Ltd.). The database search was finished with the Mascot search engine (http://www.matrixscience.co.uk) using a Mascot MS/MS ion search through National Center for Biotechnology Information nonredundant (NCBInr) databases (date: 20090410, 8201094 sequences). The parameters for searching were enzyme of trypsin; Taxonomy: Mus. (144439 sequences); Max Missed Cleavages: 1; Variable modifications of Acetyl (Protein N-term), Carbamidomethyl (C), Oxidation (M); peptide mass tolerance and fragment mass tolerance were both ± 0.2 Da.

### Western blot analysis

The extracted proteins were prepared and separated on 12% SDS-PAGE gel, and then transferred to NC membrane (Amersham Biosciences). The membrane was put into blocking buffer (5% skimmed milk in TBST) for 1 h at room temperature and blotted with primary antibodies, P2X_7_R rabbit polyclonal antibody (1:100, Santa Cruz Biotechnology, sc-25698), TCP-1 goat polyclonal antibody (1:100, Santa Cruz Biotechnology, sc-13896), in 5% skimmed milk overnight at 4°C. After washing three times for 10 min each, NC membrane was incubated with secondary antibodies, HRP-conjugated goat anti-rabbit IgG (1:5000, ZSGB-BIO), HRP-conjugated rabbit anti-goat IgG (1:5000, ZSGB-BIO), for 45 min and washed three times for 10 min each. Finally, the bands were visualized with ECL chemiluminescence detection (Beyotime). The quantity of protein loaded was verified by staining the same membranes with β-actin antibody (1:200, ZSGB-BIO). β-actin was also used to quantitatively normalize the signal.

### Immunohistochemistry (IHC)

Archival formalin-fixed paraffin-embedded sections from the 2^nd^ Affiliated Hospital of Dalian Medical University and sections of mice lymph nodes were analyzed. The sections were deparaffinized in xylene and rehydrated with a series of graded ethanol, after blocking, sections were incubated with primary TCP-1 rabbit polyclonal antibody (1:200, Bioworld technology co., BS5959), followed by HRP-conjugated anti-rabbit antibody (1:1000, ZSGB-BIO) for 30 min at 37°C. The sections were finally stained with 3,3′-diaminobenzidine (DAB) and counterstained with haematoxylin. The sections were examined independently by two pathologists who didn't know the clinicopathological information.

### Reverse transcription polymerase chain reaction

Total RNA was isolated from cells each group using TRIzol® reagent (TaKaRa Biotechnology). The sequence specificity primers was designed to amplify the coding sequence of the mouse P2X_7_R based on the recently published mouse P2X_7_R cDNA sequence by polymerase chain reaction (PCR). The primers used to detect P2X_7_R mRNA were as follows: forward primer, 5′-ATATCCACTTCCCCGGCCAC-3′, reverse primer, 5′-TCGGCAGATGGGACCAG-3′. Cycling parameters were: 94°C, 5 min for 1 cycle; 94°C, 30 sec, 57°C, 1 sec, 72°C, 1 min for 35 cycles, with extension at 72°C for 5 min. The primers used for TCP-1 were forward primer, 5′-CCTGTAAGCCTAGCCCTTTG-3′, reverse primer, 5′-CAGGGAGTTGGCTGGATAAT-3′. Cycling parameters were: 95°C, 5 min for 1 cycle; 95°C, 30 sec, 55°C, 45 sec, 72°C, 1 min for 35 cycles, with extension at 72°C for 10 min. When used as a control, the cDNA of the constitutively expressed β-actin gene was always amplified from the same cDNA preparations using specific primers, forward primer: 5′-GGCTGTATTCCCCTCCATCG-3′, reverse primer: 5′-CCAGTTGGTAACAATGCCATGT-3′. The PCR amplicon were separated in a 1.2% agarose (Pronadisa, Spain) gel containing 0.5 μg/ml ethidium bromides and visualized under UV light. Result was analyzed by Gel-Pro32 analyzer software with the values normalized to β-actin levels.

### Immunofluorescence analysis

P388D1 cells transfected with P2X_7_R shRNA and control shRNA were blocked with 10% fetal bovine serum, respectively incubated with P2X_7_R rabbit polyclonal antibody and TCP-1 goat polyclonal antibody (Santa Cruz Biotechnology), then incubated with goat anti-rabbit IgG-TRITC and rabbit anti-goat IgG-TRITC. The control shRNA group P388D1 cells was used as control.

### Statistical analyses

Statistical analyses were performed with GraphPad Prism 6 software. Student's *t*-test, Fisher exact, and Kaplan-Meier test were used as appropriate.

## SUPPLEMENTARY TABLES


