# Supplementary material for: Silencing P2X7 receptor downregulates the expression of TCP-1 involved in lymphoma lymphatic metastasis
Source: Oncotarget. 2015 Nov 6;6(39):42105–17. doi: 10.18632/oncotarget.5870 (PMC4747213; doi:10.18632/oncotarget.5870)
Supplement: Supplementary file 1 [file oncotarget-06-42105-s001.pdf]

SUPPLEMENTARY TABLES

Supplementary Table S1: Proteins identified results by mass spectrometry, Related to Results. Proteins identified by mass spectrometry as significantly changed in expression between the lymph nodes from DBA/2 mice of $P2X_7R$ shRNA groups and control groups

Spot No.	Accession	Protein Name	Gene Name	Theoretical Mrc	Theoretical Pi	Coverage(%)	Score
1	NP_598738	transferrin	Trf	76674	6.94	56%	1598
2	DAA04930	TPA: TPA_exp: keratin Kb40	Krt78	85186	8.66	1%	169
3	CAA31278	unnamed protein product	Krt8	54415	5.5	4%	87
4	NP_808385	hypothetical protein LOC239673	Krt33a			3%	68
5	BAE37790	unnamed protein product	Pgk1	44508	8.02	60%	830
6	AAA39920	testis-specific phosphoglycerate kinase	pgk-2	44882	6.63	13%	249
7	AAH08559	Unknown (protein for IMAGE:3592890)	Trf	69104	6.38	3%	52
8	NP_862897	fibrinogen beta chain	Fgb	54718	6.68	63%	1411
9	NP_058576	keratin 35	Krt35	50530	4.9		72
10	AAD01692	hair keratin basic 5; keratin Hb5	Hb5	55736	6.82	4%	65
11	NP_001020559	hypothetical protein LOC433182	Gm5506	47111	6.37	68%	1461
12	NP_031959	enolase 3, beta muscle isoform 1	Eno3	46995	6.73	16%	413
13	NP_038537	enolase 2, gamma neuronal	Eno2	47267	4.99	12%	206
14	BAB30848	unnamed protein product	Nup85	74629	5.33	1%	40
15	NP_064393.1	FK506 binding protein 12-rapamycin associated protein 1	Frap1	288789			38
16	NP_001074753	predicted gene, OTTMUSG00000008003	Gm12695	62401	9.4	1%	38
17	P23492	RecName: Full=Purine nucleoside phosphorylase	Np	32256	5.78	61%	1075
18	AAA37554	muscle-specific enolase beta subunit	Eno3	40680	6.45	3%	106
19	AAH04017	EG433182 protein	EG433182	38276	5.6	4%	106
20	AAH42515	TBC1 domain family, member 14	Tbc1d14	76765	6.01	1%	44
21	NP_058017	stress-induced phosphoprotein 1	Stip1	62542	6.4	50%	926

(*Continued*)

Spot No.	Accession	Protein Name	Gene Name	Theoretical Mrc	Theoretical Pi	Coverage(%)	Score
22	AAH27206	Heterogeneous nuclear ribonucleoprotein L	Hnrpl	60085	6.65	1%	48
23	NP_033968	chaperonin containing Tcp1, subunit 6a	Cct6a	57968	6.63	34%	461
24	Q61390	RecName: Full=T-complex protein 1 subunit zeta-2	Cct6b	58148	6.96	9%	238
25	AAD01692	hair keratin basic 5	Hb5	55736	6.82	20%	255
26	NP_081839	keratin 34	Krt34	44531	4.76	14%	249
27	XP_001471739	PREDICTED: hypothetical protein isoform 1	LOC100044179	37808	5.02	27%	246
28	CAA67580	type II intermediate filament of hair keratin	mHb6	50292	5.39	20%	238
29	NP_001003668.1	keratin 83	Krt83	54629	5.85		225
30	AAA39273	keratin type II	5430421N21Rik	54868	6.13	17%	216
31	P43277	RecName: Full=Histone H1.3; AltName	Hist1 h1d	22086	11.03	15%	193
32	AAA39372	keratin type I	Krt31	47082	4.9	12%	164
33	CAA53305	MHa3 (keratin acidic 3)	mHa3	36925	4.75	16%	152
34	BAB26243	unnamed protein product	Krt33a	46064	4.76	10%	140
35	EDL04040	mCG140891		53029	5.78	13%	126
36	NP_444479.1	keratin 82	Krt82	57140	5.99		113
37	AAA50377	spermatid-specific	His2b	15099	10.36	17%	50
38	NP_783595	histone cluster 1, H2bb	Hist1 h2bb	13944	10.31	18%	50
39	NP_034802	keratin associated protein 6–1	Krtap6–1	7999	8.33	19%	48
40	BAC98119	mKIAA1204 protein	mKIAA1204	110376	5.02	0%	44
41	NP_084474	GINS complex subunit 3	Gins3	24561	5.45	3%	41
42	AAI19602	Krt78 protein	Krt78	54730	5.9	2%	169
43	AAA37551	EndoA' cytokeratin (5′ end put.); putative		53210	5.42	4%	87
44	AAA37548	keratin type II	EndoA	54220	5.4	4%	87
45	NP_112447	keratin complex 2, basic, gene 8	Krt8	54531	5.7	4%	87
46	AAI18025	RIKEN cDNA 4732456N10 gene	4732456N10Rik	58204	8.47	3%	68
47	NP_032854	phosphoglycerate kinase 1	Pgk1	44522	8.02	60%	830

(*Continued*)

Spot No.	Accession	Protein Name	Gene Name	Theoretical Mrc	Theoretical Pi	Coverage(%)	Score
48	P09041	RecName: Full=Phosphoglycerate kinase 2	Pgk2	44854	6.36	13%	249
49	2P9Q_A	Chain A, Crystal Structure Of Phosphoglycerate Kinase-2		44751	6.7	13%	249
50	Q497I4	RecName: Full=Keratin	Krt35	50497	4.9	2%	72
51	NP_997650	keratin 73	Krt73	58875	8.36	3%	65
52	CAJ18401	Eno3	Eno3	46968	6.29	16%	413
53	EDL12600	enolase 3, beta muscle, isoform CRA_a	Eno3	48559	6.84	16%	413
54	AAH56611	Eno1 protein	Eno1	39757	5.86	4%	106
55	AAH86316	Tbc1d14 protein	Tbc1d14	68388	6.21	1%	44
56	EDL24109	heterogeneous nuclear ribonucleoprotein L, isoform CRA_b	Hnrpl	62507	7.7	1%	48
57	NP_058575	keratin complex 2, basic gene 18	Krt85	55723	6.19	20%	255
58	NP_034797	keratin 86	Krt86	53217	5.63	19%	238
59	NP_056601	histone cluster 1, H1c	Hist1 h1c	21254	11	16%	193
60	NP_034789	keratin complex 1, acidic, gene 1	Krt31	47087	4.87	12%	164
61	EDL02588	mCG20503, isoform CRA_c		42307	4.92	14%	164
62	EDL04040	mCG140891		53029	5.78	13%	126
63	NP_003509	histone cluster 1, H2bg	HIST1H2BG	13898	10.31	18%	50
64	NP_001091448	predicted gene, OTTMUSG00000013203	Gm13646	14879	10.37	17%	50

Supplementary Table S2: Patient information of 72 cases lymphoma, Related to Experimental Procedures, the detail information about patients

NO.	Age	Gender	Histologic subtypes
**72 cases lymphoma**			
1	36	Female	T-cell lymphoma
2	28	Female	Hodgkin's lymphoma
3	47	Female	B-cell lymphoma
4	53	Female	B-cell lymphoma
5	72	Male	B-cell lymphoma
6	66	Female	Diffuse large B-cell lymphoma
7	72	Female	Small lymphocytic lymphoma
8	64	Male	Diffuse large B-cell lymphoma
9	57	Male	Diffuse large B-cell lymphoma
10	50	Female	Diffuse large B-cell lymphoma
11	68	Female	B-cell lymphoma
12	78	Female	Diffuse large B-cell lymphoma
13	74	Male	Diffuse large B-cell lymphoma
14	35	Male	Hodgkin's lymphoma
15	77	Female	Diffuse large B-cell lymphoma
16	66	Male	T-cell lymphoma
17	64	Female	Diffuse large B-cell lymphoma
18	48	Female	B-cell lymphoma
19	27	Male	B-cell lymphoma
20	73	Female	T-cell lymphoma
21	23	Male	B-cell lymphoma
22	65	Male	T-cell lymphoma
23	23	Male	Hodgkin's lymphoma
24	81	Male	Diffuse large B-cell lymphoma
25	54	Male	Diffuse large B-cell lymphoma
26	54	Male	Hodgkin's lymphoma
27	34	Female	Hodgkin's lymphoma
28	53	Female	B-cell lymphoma
29	48	Male	Diffuse large B-cell lymphoma
30	45	Male	Follicular lymphoma
31	58	Male	T-cell lymphoma
32	61	Male	B-cell lymphoma
33	67	Female	Follicular lymphoma
34	75	Male	Diffuse large B-cell lymphoma
35	53	Female	Diffuse large B-cell lymphoma

(Continued)

NO.	Age	Gender	Histologic subtypes
36	58	Female	Follicular lymphoma
37	70	Male	B-cell lymphoma
38	55	Female	T-cell lymphoma
39	52	Male	Diffuse large B-cell lymphoma
40	38	Male	T-cell lymphoma
41	60	Female	Follicular lymphoma
42	20	Male	T-cell lymphoma
43	56	Male	B-cell lymphoma
44	73	Female	Hodgkin's lymphoma
45	41	Female	B-cell lymphoma
46	55	Male	Follicular lymphoma
47	64	Male	B-cell lymphoma
48	56	Male	Diffuse large B-cell lymphoma
49	58	Female	Follicular lymphoma
50	33	Female	Diffuse large B-cell lymphoma
51	43	Male	Diffuse large B-cell lymphoma
52	36	Female	Follicular lymphoma
53	50	Male	B-cell lymphoma
54	60	Female	Diffuse large B-cell lymphoma
55	53	Male	B-cell lymphoma
56	68	Male	Follicular lymphoma
57	55	Female	T-cell lymphoma
58	65	Male	Mantle cell lymphoma
59	56	Male	Diffuse large B-cell lymphoma
60	79	Female	Diffuse large B-cell lymphoma
61	58	Male	B-cell lymphoma
62	69	Male	Diffuse large B-cell lymphoma
63	51	Male	Diffuse large B-cell lymphoma
64	72	Female	Diffuse large B-cell lymphoma
65	73	Male	Follicular lymphoma
66	69	Male	Diffuse large B-cell lymphoma
67	44	Male	Follicular lymphoma
68	72	Female	Diffuse large B-cell lymphoma
69	71	Male	Diffuse large B-cell lymphoma
70	51	Male	Diffuse large B-cell lymphoma
71	55	Male	B-cell lymphoma
72	65	Male	Diffuse large B-cell lymphoma

(Continued)

NO.	Age	Gender	Histologic subtypes
**17 cases benign lymphadenopathy**			
1	6	Male	Chronic inflammation
2	47	Female	Reactive hyperplasia
3	44	Female	Reactive hyperplasia
4	49	Female	Reactive hyperplasia
5	56	Female	Reactive hyperplasia
6	41	Male	Chronic inflammation
7	75	Male	Reactive hyperplasia
8	71	Male	Reactive hyperplasia
9	27	Female	Chronic inflammation
10	42	Female	Chronic inflammation
11	46	Female	Chronic inflammation
12	4	Male	Reactive hyperplasia
13	17	Female	Reactive hyperplasia
14	65	Male	Chronic inflammation
15	73	Male	Reactive hyperplasia
16	47	Female	Chronic inflammation
17	49	Male	Chronic inflammation
